# Novel molecular subtypes of *MET*ex14 non-small cell lung cancer with distinct biological and clinical significance

**DOI:** 10.1038/s41698-024-00642-6

**Published:** 2024-07-26

**Authors:** Shengnan Chen, Tao Hu, Jikai Zhao, Qian Zhu, Jin Wang, Zhan Huang, Chan Xiang, Ruiying Zhao, Changbin Zhu, Shun Lu, Yuchen Han

**Affiliations:** 1 Department of Pathology, Shanghai Chest Hospital, School of Medicine, Shanghai Jiaotong University, Shanghai, China; 2 Department of Medicine, Amoy Diagnostics Co., Ltd., Xiamen, China; 3 Department of Oncology, Shanghai Chest Hospital, School of Medicine, Shanghai Jiaotong University, Shanghai, China

**Keywords:** Non-small-cell lung cancer, Tumour heterogeneity

## Abstract

Not all *MET* exon 14 skipping (*MET*ex14) NSCLC patients benefited from MET inhibitors. We hypothesized an inter-tumoral heterogeneity in *MET*ex14 NSCLC. Investigations at genomic and transcriptomic level were conducted in *MET*ex14 NSCLC samples from stage I-III and recurrent/metastatic patients as discovery and validation cohort. Four molecular subtypes were discovered. MET-Driven subtype, with the worst prognosis, displayed MET overexpression, enrichment of MET-related pathways, and higher infiltration of fibroblast and regulatory T cells. Immune-Activated subtype having the most idea long-term survival, had higher tertiary lymphoid structures, spatial co-option of PD-L1^+^ cancer cells, and GZMK^+^ CD8^+^ T cell. FGFR- and Bypass-Activated subtypes displayed FGFR2 overexpression and enrichments of multiple oncogenic pathways respectively. In the validation cohort, patients with MET-Driven subtype had better response to MET inhibitors than those with MET overexpression. Thus, molecular subtypes of *MET*ex14 NSCLC with distinct biological and clinical significance may indicate more precise therapeutic strategies for *MET*ex14 NSCLC patients.

## Introduction

*MET* Exon14 skipping (*MET*ex14) is an oncogenic driver occurring in 1–3% of patients with lung adenocarcinoma (LUAD)^1–3^ and lung squamous carcinoma (LUSC)^4^. *MET*ex14 NSCLC responds to the treatment of MET tyrosine kinase inhibitors (MET-TKIs), with the reported objective response rate (ORR) ranging from 32% to 67.9% and median progression-free survival (mPFS) was from 5.4–9.7 months^5–8^. Thus, not all *MET*ex14 NSCLC patients benefited from the treatment of MET inhibitors. We hypothesized an inter-tumoral heterogeneity in ex14 NSCLC determining response of *MET*ex14 NSCLC to MET-TKIs. And it would be of clinical importance in influencing decision of management for *MET* ex14 NSCLC patients.

Regarding complex genomic locations and various types of mutations, *MET*ex14 NSCLC may putatively harbor biological heterogeneity^9,10^. Meanwhile, MET expression varied in *MET*ex14 NSCLC patients. As reported, *MET*ex14 NSCLC with MET over-expression got more benefit from MET inhibitor treatment^6,11^. On the other side, NSCLC patients with *MET*ex14 displayed higher levels of PD-L1 both at transcription and protein levels. Higher immune infiltration and IFN-γ signatures were also reported by previous studies^12,13^. However therapeutic efficacy of immunotherapy remained controversial in patients with *MET*ex14 NSCLC^14–16^. These recent translational and clinical studies strongly indicated a potential inter-heterogeneity in *MET*ex14 NSCLC.

While no systematic investigation by far has been conducted to disclose the inter-tumor heterogeneity in *MET*ex14 NSCLC. To address this hypothesis, comprehensive genomic and transcriptomic profiling was performed together with the immunophenotype of the tumor microenvironment. A better understanding of the biological and tumor microenvironmental diversity of *MET*ex14 NSCLC may provide insight into a more precise evaluation the risk of disease recurrence at the early disease stage. It may also indicate novel therapeutic approaches for advanced/metastatic NSCLC patients with *MET*ex14.

## Results

### Transcriptomic profiling reveals Intertumoral heterogeneity among *MET*ex14 NSCLC patients

Limited genomic location-specific differentially expressed genes in *MET* ex14 NSCLC were discovered (Supplementary Fig. 1), which indicated the limited impact of various genomic locations of *MET* ex14 on inter-tumoral heterogeneity.

**Fig. 1:**
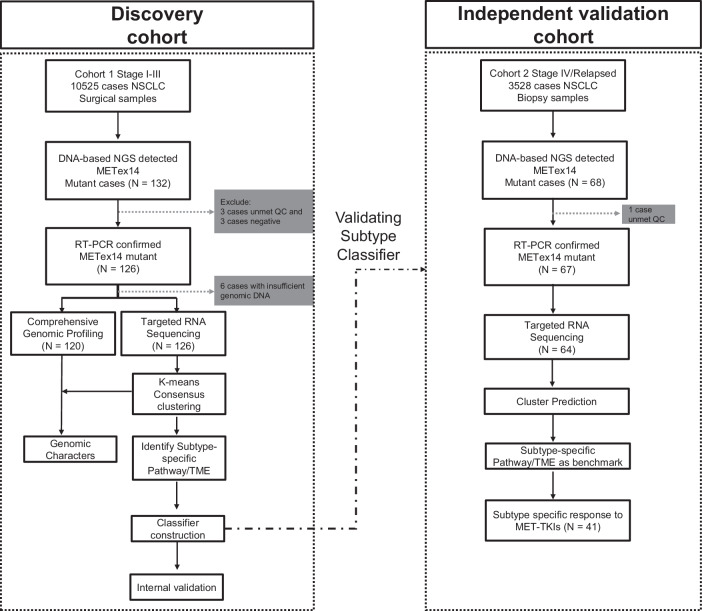
Flow diagram illustrating the patients included for the analytical process.

**Fig. 2:**
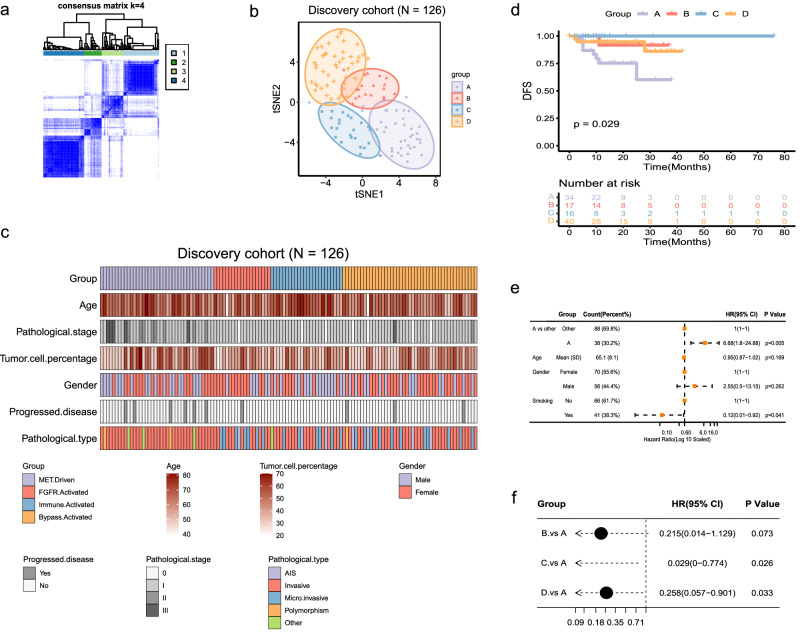
Classification of *MET*ex14 NSCLC from the discovery cohort. **a** The consensus matrix represented as heatmap for the chosen optimal cluster number (k = 4) for unsupervised clustering analysis on RNA-seq data. **b** The t-SNE plot shows the localization of each of the four tumor classes. **c** Comparison of clinical characteristics between molecular subtypes. **d** Disease-free survival of the four subtypes in the discovery cohort. Forest plots showing multivariate (**e**) and univariate (**f**) analysis of the molecular subtype and clinical risk factors associating with DFS.

Transcriptomics-based molecular subtypes were discovered revealing inter-tumoral heterogeneity in various malignant solid tumors^17–20^. In this study, targeted RNA sequencing was successfully conducted in the discovery and validation cohort with the success rate of 97.67% (126/129) and 95.52% (64/67), respectively (Fig. 1). To understand the inter-tumor heterogeneity, enrichment of pathways in KEGG, Reactome, Hallmark was evaluated in each sample utilizing RNA-seq data from discovery cohort (*n* = 126) followed by k-means clustering. As informed by consensus cumulative distribution function (CDF) and the relative change in area under the curve (AUC) for CDF, (Supplementary Fig. 2a, b), four well-distinguished subtypes (*κ* = 4) in *MET*ex14 NSCLC from the discovery cohort were discovered for the first time (Fig. 1, Fig. 2a, b), with 38 cases (30.2%) of subtype A, 19 cases (15.1%) of subtype B, 24 cases (19.0%) of subtype C and 45 cases (35.7%) of subtype D.

### Molecular subtype distinct clinic-pathological significance in *MET*ex14 NSCLC patients

Subtype A was significantly associated with clinical (*P* = 0.0023) and pathologic (*P* < 0.001) stage II/III, especially with clinical and pathological T2-3 (*P* < 0.001) (Fig. 2c, Table 1). Subtype A also displayed clinic-pathologically more aggressive characteristics, such as invasive subtype (*P* < 0.0001) and pleural invasion (*P* = 0.004) (Fig. 2c, Table 1). In 107 patients with long-term follow-up, subtype A and C had the most unfavored and best prognosis, respectively (Fig. 2d, *P* = 0.029). Of note, three-year DFS rate of subtype C reached to 100% (Fig. 2d). Multi and univariate cox regression analysis showed subtype A was independently associated with shorter DFS (hazard ratio [HR] = 6.68 [95% CI 1.80–24.88], *P* = 0.005) (Fig. 2e, f). These well-defined molecular subtypes with unique clinic-pathologic features suggested subtype-specific biological features.

**Table 1:** Baseline characteristics of stage I-III NSCLC patients with *MET*ex14 in the discovery cohort

Characteristic	Overall (*n* = 126)	A (*n* = 38)	B (*n* = 19)	C (*n* = 24)	D (*n* = 45)	*P*-value
**Gender**						
Female	70 (55.6%)	19 (50.0%)	13 (68.4%)	9 (37.5%)	29 (64.4%)	0.108
Male	56 (44.4%)	19 (50.0%)	6 (31.6%)	15 (62.5%)	16 (35.6%)	
**Age**						
Mean (SD)	65.1 (8.06)	66.4 (7.11)	62.6 (8.57)	68.7 (7.87)	63.2 (8.02)	0.026
Median [Min, Max]	66.0 [39.0, 81.0]	66.0 [55.0, 81.0]	62.0 [42.0, 76.0]	69.0 [53.0, 79.0]	64.0 [39.0, 77.0]	
**Smoking**						
Never	66 (52.4%)	21 (55.3%)	12 (63.2%)	7 (29.2%)	26 (57.8%)	0.421
Ever/Current	41 (32.5%)	13 (34.2%)	5 (26.3%)	9 (37.5%)	14 (31.1%)	
NA	19 (15.1%)	4 (10.5%)	2 (10.5%)	8 (33.3%)	5 (11.1%)	
**Tumor cell percentage**						
Mean (SD)	41.3 (11.2)	44.5 (13.3)	38.4 (9.58)	40.0 (9.78)	40.4 (10.2)	0.36
Median [Min, Max]	40.0 [20.0, 70.0]	40.0 [30.0, 70.0]	40.0 [20.0, 60.0]	40.0 [30.0, 60.0]	40.0 [30.0, 60.0]	
**Pathological stage**^**a**^						
I	111 (88.1%)	25 (65.8%)	19 (100%)	24 (100%)	43 (95.6%)	<0.001
II	9 (7.1%)	8 (21.1%)	0 (0%)	0 (0%)	1 (2.2%)	
III	6 (4.8%)	5 (13.2%)	0 (0%)	0 (0%)	1 (2.2%)	
**Pathological T**						
T1	84 (66.7%)	12 (31.6%)	16 (84.2%)	22 (91.7%)	34 (75.6%)	<0.001
T2	27 (21.4%)	18 (47.4%)	2 (10.5%)	0 (0%)	7 (15.6%)	
T3	8 (6.3%)	8 (21.1%)	0 (0%)	0 (0%)	0 (0%)	
NA	7 (5.6%)	0 (0%)	1 (5.3%)	2 (8.3%)	4 (8.9%)	
**Pathological N**						
N0	117 (92.9%)	31 (81.6%)	19 (100%)	24 (100%)	43 (95.6%)	0.153
N1	4 (3.2%)	3 (7.9%)	0 (0%)	0 (0%)	1 (2.2%)	
N2	5 (4.0%)	4 (10.5%)	0 (0%)	0 (0%)	1 (2.2%)	
**Clinical stage**^**a**^						
I	93 (73.8%)	22 (57.9%)	17 (89.5%)	16 (66.7%)	38 (84.4%)	0.004
II	8 (6.3%)	7 (18.4%)	0 (0%)	0 (0%)	1 (2.2%)	
III	6 (4.8%)	5 (13.2%)	0 (0%)	0 (0%)	1 (2.2%)	
NA	19 (15.1%)	4 (10.5%)	2 (10.5%)	8 (33.3%)	5 (11.1%)	
**Clinical T**						
T1	68 (54.0%)	11 (28.9%)	15 (78.9%)	14 (58.3%)	28 (62.2%)	<0.001
T2	27 (21.4%)	17 (44.7%)	1 (5.3%)	0 (0%)	9 (20.0%)	
T3	6 (4.8%)	6 (15.8%)	0 (0%)	0 (0%)	0 (0%)	
NA	25 (19.8%)	4 (10.5%)	3 (15.8%)	10 (41.7%)	8 (17.8%)	
**Clinical N**						
N0	98 (77.8%)	27 (71.1%)	17 (89.5%)	16 (66.7%)	38 (84.4%)	0.226
N1	4 (3.2%)	3 (7.9%)	0 (0%)	0 (0%)	1 (2.2%)	
N2	5 (4.0%)	4 (10.5%)	0 (0%)	0 (0%)	1 (2.2%)	
NA	19 (15.1%)	4 (10.5%)	2 (10.5%)	8 (33.3%)	5 (11.1%)	
**Pathological type**						
Invasive	88 (69.8%)	33 (86.8%)	11 (57.9%)	15 (62.5%)	29 (64.4%)	0.0015
Microinvasive	24 (19.0%)	0 (0%)	7 (36.8%)	6 (25.0%)	11 (24.4%)	
Polymorphism	3 (2.4%)	2 (5.3%)	0 (0%)	0 (0%)	1 (2.2%)	
AIS	7 (5.6%)	0 (0%)	1 (5.3%)	2 (8.3%)	4 (8.9%)	
Other	4 (3.2%)	3 (7.9%)	0 (0%)	1 (4.2%)	0 (0%)	
**Acinar**						
≤50%	52 (41.3%)	20 (52.6%)	4 (21.1%)	9 (37.5%)	19 (42.2%)	0.375
>50%	34 (27.0%)	11 (28.9%)	7 (36.8%)	6 (25.0%)	10 (22.2%)	
NA	40 (31.7%)	7 (18.4%)	8 (42.1%)	9 (37.5%)	16 (35.6%)	
**Papillary**						
≤50%	24 (19.0%)	13 (34.2%)	1 (5.3%)	5 (20.8%)	5 (11.1%)	0.136
>50%	4 (3.2%)	1 (2.6%)	1 (5.3%)	2 (8.3%)	0 (0%)	
NA	98 (77.8%)	24 (63.2%)	17 (89.5%)	17 (70.8%)	40 (88.9%)	
**Micropapillary**						
≤50%	12 (9.5%)	7 (18.4%)	1 (5.3%)	1 (4.2%)	3 (6.7%)	0.454
>50%	1 (0.8%)	0 (0%)	0 (0%)	0 (0%)	1 (2.2%)	
NA	113 (89.7%)	31 (81.6%)	18 (94.7%)	23 (95.8%)	41 (91.1%)	
**Solid**						
≤50%	12 (9.5%)	7 (18.4%)	1 (5.3%)	0 (0%)	4 (8.9%)	0.175
>50%	8 (6.3%)	8 (21.1%)	0 (0%)	0 (0%)	0 (0%)	
NA	106 (84.1%)	23 (60.5%)	18 (94.7%)	24 (100%)	41 (91.1%)	
**Lepidic**						
≤50%	39 (31.0%)	12 (31.6%)	7 (36.8%)	12 (50.0%)	8 (17.8%)	0.005
>50%	25 (19.8%)	4 (10.5%)	3 (15.8%)	2 (8.3%)	16 (35.6%)	
NA	62 (49.2%)	22 (57.9%)	9 (47.4%)	10 (41.7%)	21 (46.7%)	
**Pleural invasion**						
PL0	106 (84.1%)	23 (60.5%)	19 (100%)	23 (95.8%)	41 (91.1%)	0.004
PL1	10 (7.9%)	6 (15.8%)	0 (0%)	1 (4.2%)	3 (6.7%)	
PL2	8 (6.3%)	7 (18.4%)	0 (0%)	0 (0%)	1 (2.2%)	
PL3	2 (1.6%)	2 (5.3%)	0 (0%)	0 (0%)	0 (0%)	

*NA* not applicable, *NSCLC* Non-Small Cell Lung Cancer.

^**a**^American Joint Committee on Cancer (AJCC) 8th.

### Comprehensive genomic profiling displayed a relatively well-balanced subtype-specific genomic characteristics

To explore subtype-specific genomic characteristics, comprehensive genomic profiling was conducted and analyzed. *MET* amplification (~5% in 120 samples) was balanced across four subtypes (Supplementary Fig. 3a). *TP53*, which had a mutation frequency of 31.6% in subtype A and was rarer in the other subtypes (Supplementary Fig. 3a). No association between *TP53* mutations and prognosis was observed, either across the cohort or only within subtype A (Supplementary Fig. 3b, c). *FGF19*, *AKT2*, and *FGF3* amplifications were significantly higher in subtype A; *FGF3*, *PDCD1*, and *FGF19* deletions were more commonly occurred in subtype B and D (Supplementary Fig. 3a). No association was found between these CNV gain/loss and prognosis, mRNA expression, tumor microenvironment features, signaling pathways enrichment (Supplementary Fig. 4a–c). No significantly distributed specific mutation signature was found across four subtypes (Supplementary Fig. 3a). No differential distribution of genomic locations of *MET* ex14 variants was observed across these four subtypes (Supplementary Fig. 3d, e).

### Unique activated oncogenic pathways in four molecular subtypes

**Fig. 3:**
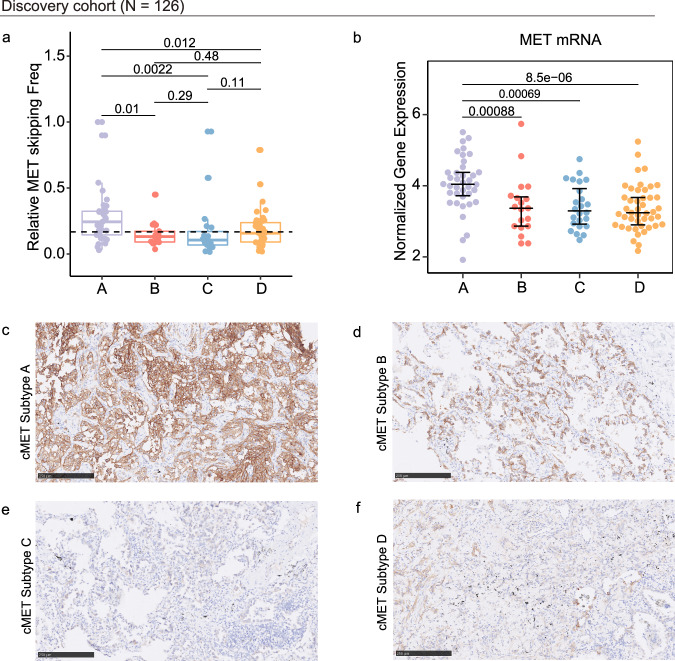
Analysis of *MET* mutation frequency, and the expression of MET in the discovery cohort. **a** *MET* exon 14 skipping mutation frequency in each subtype. **b** Expression of *MET* mRNA in the four subtypes. **c**–**f** Representative example of MET immunohistochemical staining result of each subtype.

**Fig. 4:**
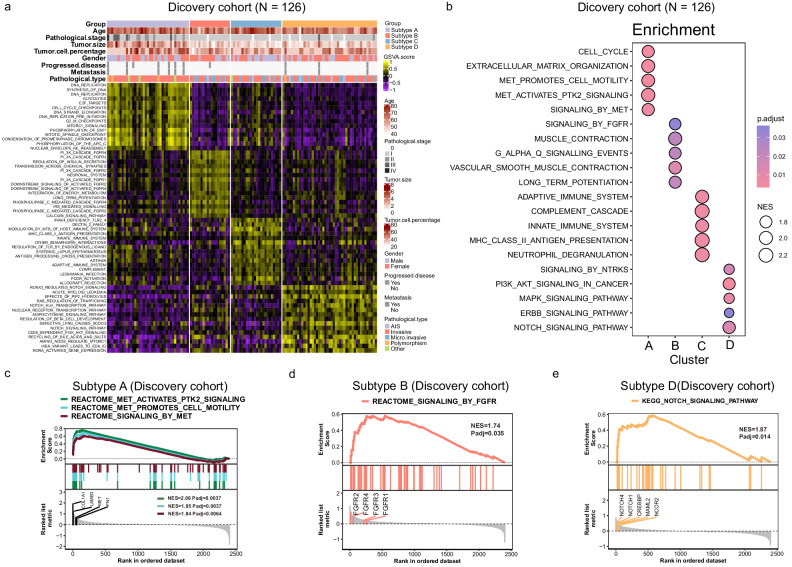
Gene expression profile of the four molecular subtypes from the discovery cohort. **a** GSVA analysis of differentially expressed genes in the four subtypes based on the curated KEGG, Reactome, and Hallmark gene sets. **b** ssGSEA analysis of differentially expressed genes based on the KEGG gene set. **c** ssGSEA result showed that COL1A1, LAMB3, MET, and FN1 were significantly enriched in three pathways “MET activates PTK2 signaling”, “MET promotes cell motility”, and “signaling by MET” in subtype A. **d** ssGSEA result showed that FGFR2, FGFR4, FGFR3, and FGFR1 were significantly enriched in “SIGNALING_BY_FGFR” pathway in subtype B. **e** ssGSEA result showed that NOTCH4, NOTCH1, CREBBP, MAML2, and NCOR2 were significantly enriched in “NOTCH_SIGNALING_PATHWAY” pathway in subtype D.

Variant allele frequency (VAF) of *MET*ex14 was highest in subtype A (Fig. 3a, *P* = 0.0022), together with *MET* gene and c-MET protein over expression (Fig. 3b–f, *P* < 0.001). Pathways mostly enriched in subtype A were associated with promoting cell-cycle, DNA replication and MET-driven cell motility and PTK2 signaling (Fig. 4a–c).

Subtype B was mostly related to FGFR pathways, such as PI3K_CASCADE_FGFR1-4, and DOWTREAM SIGNALING OF ACTIVATED FGFR 1-4 (Fig. 4a, b). And Subtype B was characterized by *FGFR2* over-expression (Fig. 4d).

Subtype C was characterized by the enrichment of immune-related pathways, such as MHC_CLASS_II_ANTIGEN, INNATE IMMUNE SYSTEM, and ADAPTIVE IMMUNE SYSTEM (Fig. 4a, b).

Activation of multiple signaling pathways, such as *NOTCH*, *FGFR*, *PIP2*, *PI3K-AKT*, *ERBB2*, and *IKBA* were found in subtype D (Fig. 4a, b). Further analysis of the “NOTCH_SIGNALING_PATHWAY” pathway revealed that *NOTCH4*, *NOTCH1*, *CREBBP*, *MAML2*, and *NCOR2* were highly expressed (Fig. 4e).

### Distinct tumor immune microenvironment (TME) in four molecular subtypes

**Fig. 5:**
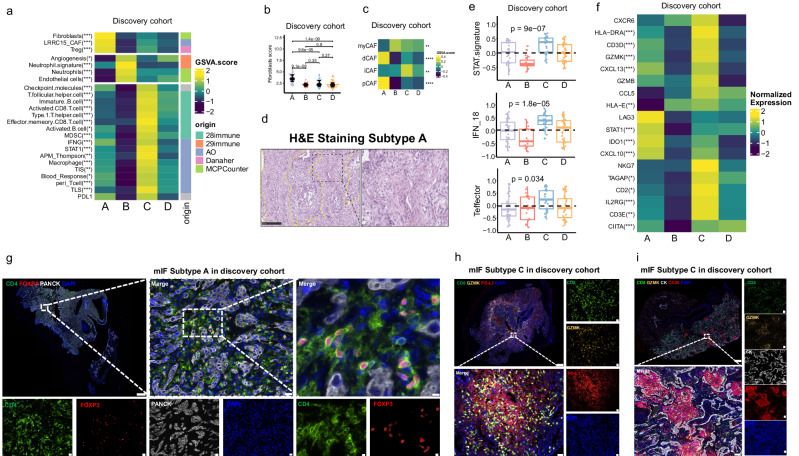
Tumor microenvironment of the four molecular subtypes from the discovery cohort. **a** Signatures from 28 immunity, 29 immunity, AO, Danaher and MCPCounter reference sets were found to hold significant differences among four subtypes. **b** Expression of four cancer-associated fibroblasts (CAF) signatures, myofibroblastic CAF (myCAF), development CAF (dCAF), inflammatory CAF (iCAF) and pCAF, and **c** fibroblasts score in the four subtypes. **d** A representative H&E staining of subtype A. **e** Expression of STAT signature, IFN-γ related genes, and effector T cell gene signature of the four subtypes. **f** Expression of 18 genes related to IFN-γ signature in the four subtypes. **g** Representative example of multiple immunofluorescences of Treg cells of subtype A. **h** Representative example of multiple immunofluorescences of effector CD8^ +^ T cell of subtype C. **i** Representative example of multiple immunofluorescences of CD20^ +^ B cell of subtype C.*, **, and *** represent *P* < 0.05, *P* < 0.01, and *P* < 0.001, respectively.

Part of *MET*ex14 NSCLC patients benefited from immune checkpoint inhibitors^21^, but others did not^22,23^, which suggested heterogenous tumor microenvironment in *MET*ex14 NSCLC. As discovered, each molecular subtype displayed a distinct TME phenotype (Fig. 5a, Supplementary Fig. 5a–e). Subtype A displayed highly immunosuppressive TME characterized by infiltration of regulatory T (Treg) cells and PDCD1 (PD-1) expression (Fig. 5a, b, Supplementary Fig. 5f, Supplementary Fig. 6a). Meanwhile highly enriched cancer-associated fibroblasts (CAF) signatures can also be observed in subtype A tumors, especially for pan-CAF (pCAF), desmoplastic CAF (dCAF) and LRRC15 ^+^ CAF (Fig. 5c). Histologically, condensed desmoplastic phenotype and fibrosis in H&E staining further supported the findings of enriched CAF signatures in subtype A (Fig. 5d).

On the other hand, anti-tumor immunity was more prominent in subtype C, with significantly elevated enrichment of Effector cells, T cells, Th1 signature, B cell, Coactivation molecules (Supplementary Fig. 5a–e, Supplementary Fig. 6b), and increased level of inflammatory cancer-associated fibroblast (iCAF) (Fig. 5c). Moreover, pro-inflammatory signatures like STAT signature, IFN-γ signature, and effector T cell signature were also significantly enriched in Subtype C compared with other subtypes (Fig. 4e). Of note, IFN-γ related genes was also highly upregulated in subtype C (Fig. 4f).

In parallel, mIF showed an aggregation of CD4^+^/FOXP3^+^ Treg cells in tumor tissues of subtype A (Fig. 5g), but almost invisible in the tumor tissue of the other three subtypes (Supplementary Fig. 7). In subtype C, a larger number of infiltrated effector CD8 ^+^ T cells (CD8^+^/GZMK^+^) and spatial co-option with PD-L1^+^ cancer cells (Fig. 5h) were observed compared to the other three subtypes (Supplementary Fig. 8). Higher number of tertiary lymphoid structures (TLS) characterized by accumulated CD20^+^ B cells were also detected in subtype C (Fig. 5i, Supplementary Fig. 9). These findings proved that in *MET*ex14 NSCLC from the discovery cohort, patients with immune enriched subtype (subtype C) potentially benefited from ICIs, while patients with subtype A, prominent fibrosis and Treg infiltration, would putatively be resistant to ICIs.

### Constructing subtype classifier and predicting molecular subtypes in relapsed/metastatic *MET* ex14 NSCLC patients

**Fig. 6:**
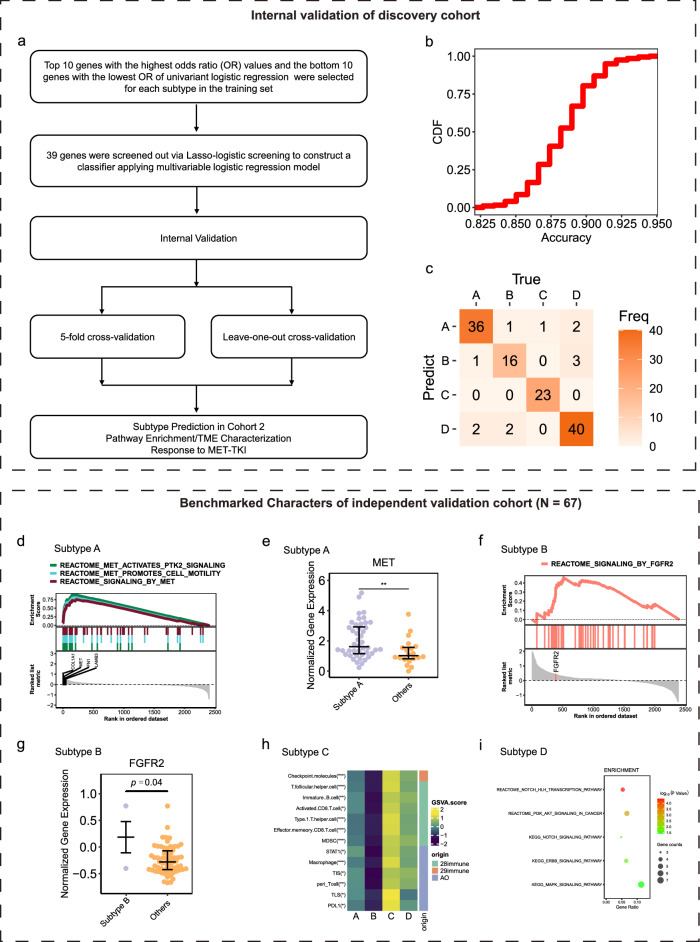
A prediction model constructed with stage I-III patients (the discovery cohort) and evaluated in patients with relapsed/metastatic disease (the independent validation cohort). **a** Schematic diagram of model construction process. **b** The predictive performance in 5-fold cross-validation of the prediction model based on (the discovery cohort). **c** Leave-one-out cross-validation showing the model prediction results of the discovery cohort. **d** ssGSEA result showed that COL1A1, MET, FN1, and LAMB3 were significantly enriched in three pathways “MET activates PTK2 signaling”, “MET promotes cell motility”, and “signaling by MET” in subtype A in the independent validation cohort. **e** Expression of *MET* gene in the four subtypes in the independent validation cohort. **f** ssGSEA result showed that FGFR2 was significantly enriched in “SIGNALING_BY_FGFR2” pathway in subtype B in the independent validation cohort. **g** Expression of *FGFR2* gene in the independent validation cohort. **h** Signatures from 28 immunity, 29 immunity, AO, Danaher, and MCPCounter with significant differences among four subtypes in the independent validation cohort. **i** ssGSEA result showed that differentially expressed genes were enriched in NOTCH, PI3K-AKT, ERBB2, and MAPK SIGNALING pathways in subtype D in the independent validation cohort. *, **, and *** represent *P* < 0.05, *P* < 0.01, and *P* < 0.001, respectively.

To translate biological diversity of *MET*ex14 NSCLC into a clinically feasible tool, a subtype classifier was constructed based on the transcriptomic data in the discovery cohort (Fig. 6a). Previous studies in cancers such as SCLC^17^, CRC^18,24^, and breast cancer^19,25,26^ have demonstrated the feasibility of using early-stage patients for subtype identification and then validation in advanced patients. An independent cohort of relapsed/metastatic NSCLC patients was enrolled (Fig. 1) to verify the performance of the subtype classifier. To ensure the biological comparability, differences in gene expression, tumor microenvironment, and signaling pathways enrichment between the discovery cohort and validation cohort patients were analyzed by tSNE methods. Overall, a well-balanced expression profile was found between early- and late-stage NSCLC with *MET*ex14 mutations (Supplementary Fig. 3f–h).

Briefly, 20 leading genes were screened for each subtype using univariant logistic regression, and a total of 80 genes were obtained (Supplementary Fig. 10a). 39 of 80 genes were selected by lasso-logistic regression for classifier construction (Supplementary Fig. 10b). Subtype-specific genes in each group were also selected (Supplementary Fig. 10c). Five-fold cross-validation and LOOV showed with more than 80% accuracy (Figs. 6b), and 90.5% of overall concordance rate in LOOV (Fig. 6c) respectively.

In the validation cohort, four subtypes were predicted and showed well-balanced clinic-pathological characteristics (Supplementary Table 1). Benchmarked MET-related signaling pathways were also enriched in predicted subtype A (MET-Driven) (68.8%) (Fig. 6d), with the expression of *MET* genes was significantly higher in this subtype (Fig. 6e). A tendency for FGFR-related signaling pathways was observed to be enriched in predicted subtype B (FGFR Activated) (Fig. 6f), and *FGFR2* gene expression was significantly upregulated (Fig. 6g). TME phenotype of each subtype in this validation set similarly reveal that predicted subtype C (Immune-Activated) (21.9%) was immunologically activated (Fig. 6h). And activation of NOTCH, PI3K-AKT, ERBB2 and MAPK were observed in predicted subtype D (Bypass-Activated) (Fig. 6i).

Of note, one patient (a 68-year-old female, non-smoker) with Immune-Activated subtype in the validation cohort was treated with immune-chemo combined therapy (nivolumab + pemetrexed + carboplatin) and achieved a partial response with a PFS of 16 months (Supplementary Fig. 11).

### MET-Driven patients displayed better responses to MET-targeting tyrosine kinase inhibitors

**Fig. 7:**
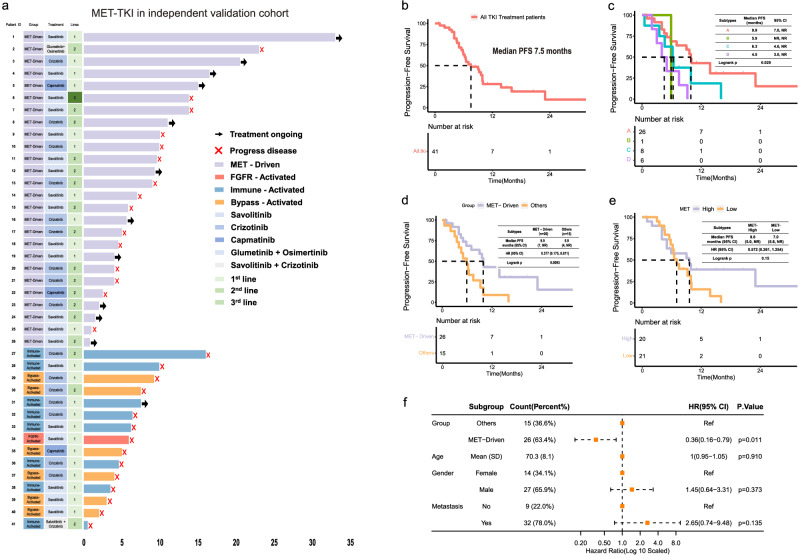
Association of molecular subtype with patient prognosis and response to MET-TKI in the independent validation cohort. **a** Swimmer plot showed the follow-up information of the 41 patients treated with MET-TKI in a time range of 35 months. **b** Kaplan-Meier plot showed progression-free survival (PFS) of all the 41 patients treated with MET-TKI. **c** PFS of the patients who received MET-TKI treatment with four predicted subtypes. **d** PFS of the predicted subtype A patients and patients with the other three predicted subtypes received MET-TKI treatment. **e** PFS of the patients received MET-TKI treatment with high or low *MET* expression. **f** Forest plot of showing multivariate COX regression of the molecular subtype and clinical risk factors associated with PFS.

To understand the drug vulnerability of each subtype of *MET*ex14 NSCLC, the survival of 41 patients in the independent validation cohort with the treatment of MET inhibitors was analyzed (Fig. 7a). 26 patients were MET-Driven subtype (subtype A) and 15 patients belonged to other subtypes. Baseline characteristics were comparable between patients with MET-Driven subtype and those with the other three subtypes (Supplementary Table 2). The follow-up cut-off was June 2023. The median progression-free survival (PFS) of the 41 patients was 7.5 months (Fig. 7b). Patients with MET-Driven subtype responded better to MET inhibitors and displayed longer PFS than the other three predicted subtypes (median PFS: 9.9 months vs 5.9 months vs 6.3 months vs 4.5 months, *P* = 0.029, Fig. 7 a, c), (median PFS, 95%CI: 9.9 months,7-NR vs 5.9 months, 4-NR; HR, 95%CI: 0.377, 0.175–0.811, *P* = 0.0093, Fig. 7d). Moreover, MET-Driven subtype independently associated with the benefit of MET inhibitors (HR, 95%CI: 0.36, 0.16–0.79, *P* = 0.011, Fig. 7f).

However, PFS was not significantly different for patients with high *MET* expression and those with low *MET* expression (median PFS: 9.6 months vs 7.0 months; HR,95%CI: 0.572, 0.261–1.254, *P* = 0.15, Fig. 7e). These results suggested MET inhibitors may be used more precisely, especially to those with MET-Driven subtype.

## Discussion

Short-term response to MET inhibitors observed in multiple clinical trials as well as dis-concordant efficacy of immunotherapy leads to the following hypothesis: there is an inter-patient/tumor heterogeneity with clinical and biological significance in patients with *MET*ex14 NSCLC^21^. As suggested by this study, diverse genomic locations, or types of *MET*ex14 variants are not directly associated with inter-tumoral heterogeneity. In parallel, recent clinical trials of MET-TKIs suggested there is no direct association between the locations or type of the *MET*ex14 alterations and therapeutic efficacy of the targeted MET inhibitor^6,27^.

**Fig. 8:**
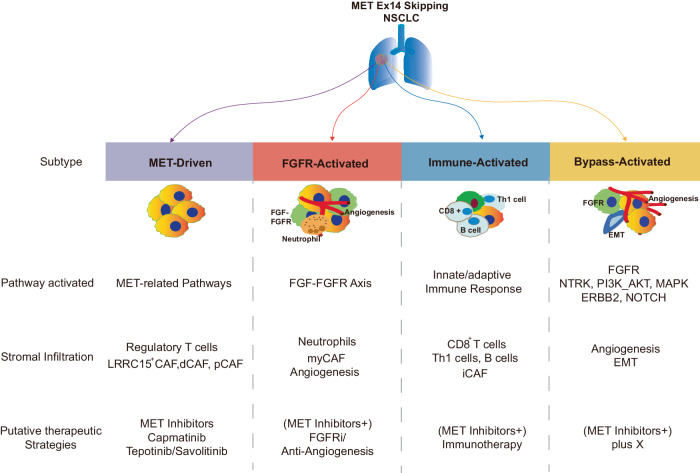
Diagram illustrating the molecular characteristics, clinicopathologic features, and prognostic indications of each subtype.

In this study, four subtypes (MET-Driven, FGFR-Activated, Immune-Activated and Bypass-Activated) with differentially clinic-pathological characteristics, prognosis and drug vulnerability were discovered for the first time at transcriptional level (Fig. 8). Co-occurred genomic alterations either *TP53* or copy number variations had limited impact on clinical and biological characterizations of *MET* ex 14 NSCLC. MET-Drive subtype (30.2% in stage I-III patients; 68.8% in relapsed/metastatic patients), showed higher VAF of *MET*ex14 together with overexpression of *MET* gene, c-MET protein, and enrichment of MET-related pathways. As reported, the frequency of *MET*ex14 and expression of MET correlated with the efficacies of MET-TKIs^5,27^. Parallelly, MET-Driven patients were significantly vulnerable to MET-TKIs and displayed longer PFS than other subtypes.

MET-Driven tumors displayed an immunosuppressive phenotype with infiltration of Tregs and LRRC15^+^ and desmoplastic fibroblasts. High expression of the LRRC15^+^ CAF and desmoplastic CAFs signature was associated with poor response to immune-checkpoint blockade in several different human tumor types^28,29^. As such, even in MET-Driven tumors, PD-L1, as well as other inhibitory immune checkpoint molecules^21,30^ were upregulated in tumor cells, this immunosuppressive and exclusive TME would attenuate the efficacy of immunotherapy^31^. These findings may provide a preliminary explanation of the paradox in clinics of why significant proportions of PD-L1 positive *MET*ex14 NSCLC patients fail to respond to PD-1/PD-L1 treatment.

Oppositely, Immune-Activated subtype (19.0% in stage I-III patients; 21.9% in relapsed/metastatic patients) were characterized by significant enrichment of proinflammatory signatures like IFN-γ, effector T cells and inflammatory CAFs. Immunofluorescence further suggested increased infiltrated CD8^+^/GZMK^+^ T cells and TLS in immune-activated subtype. It can be inferred that patients with immune activated phenotype may benefit from immunotherapy. Recent studies reported portions of NSCLC patients harboring *MET*ex14 alterations displayed durable response to immunotherapy, which supported this hypothesis. Particularly, partial, or complete responses over more than 18 months were seen in six patients (46.2%) with METex14 alterations^32^. In addition, four NSCLC patients with *MET*ex14 and *MET* amplification had long-lasting response to second- or late line immunotherapy^12^. Immune-Activated subtype may define a subset of METex14 NSCLC patients may benefit from immunotherapy or a combination of immune checkpoint inhibitors with MET-TKIs.

FGFR-Activated subtype (15.1% in stage I-III patients; 3.1% in relapsed/metastatic patients) in *MET*ex14 NSCLC was mainly characterized by *FGFR*1-4 overexpression, especially *FGFR2* overexpression, and enrichment of *FGFR* signaling pathways. Previous studies have found that activation of the FGFs-FGFRs pathways contributes to NSCLC pathogenesis and progression via autocrine and paracrine loops^33,34^. Cancer-associated fibroblast with myofibroblast differentiation also displayed activation of FGF-FGFR pathways mediating drug resistance^35^. Activation of these pathways may trigger resistance of multiple therapeutic modalities like anti-MET^36^, anti-EGFR^37^, and anti-vascular endothelial growth factor (VEGF)^38^ therapies. As suggested by a recent investigation on FGFR1-4 over-expressed breast cancer, FGFR inhibitor can cause shrinkage of tumors with elevation of FGFR1-4 mRNA^39^. Thus, the potential application of FGFR inhibitors might be considered as a combinational strategy with MET inhibitors for FGFR-overexpressed patients with *MET*ex14.

Bypass subtype, featuring enrichment of multiple oncogenic pathways, including NTRK, NOTCH, ERBB2, PI3K-AKT and MAPK pathways, may confer therapeutic resistance to MET tyrosine kinase inhibitors^40–42^. Biologically, *MET*ex14 could lead to trans-activation of multiple intracellular signaling pathways including RAS-MAPK^43^, SMAD2^44^ resulting tumor progression and resistant to MET-targeted therapies. And, it is scientifically rational that there is mixture of genetic and non-genetic clones in malignant tumors^45^. And the mixture of various pathway-dominated clones promotes cancer progression^46^. Clinically, among the MET-TKI treated patients in this study, Bypass-Activated patients displayed the most inferior response to MET-TKI, with significantly shorter mPFS. Thus, it can be inferred that *MET*ex14 NSCLC patients in Bypass-Activated subtype may not benefit from either MET-TKI or immunotherapy, and instead require additional investigation to identify alternative options of combinational strategy.

Regarding the unbalanced proportion of FGFR-Activated and Bypass-Activated subtypes between the discovery and validation cohort, this discrepancy may be explained by the clone evolution hypothesis during cancer progression. Activation of FGF-FGFR or WNT, ERBB2, MAPK pathways may promote oncogenesis from preinvasive to invasive stage^47–49^. Based on the results from this study, it can be hypothesized that there was co-occurrence of clones with *MET*ex14 and non-genetic subclone with FGFR2 overexpression (FGFR-Activated subtype) or by WNT, ERBB2, MAPK pathways (Bypass-Activated subtype) in part of *MET*ex14 NSCLC. During cancer progression, majority of cancer cells were putatively clones of minority by *MET* exon 14 clones, with diminished clones with FGF-FGFR or WNT, ERBB2, MAPK pathways, which may lead less FGFR-Activated or Bypass-Activated subtype in more advanced disease stages. This hypothesis deserves in-depth investigation and further validation in a larger sample size of patients.

This study has several limitations. Firstly, most of the patients were enrolled at early disease stages, with a larger majority of patients in stage I (111 of 126, 88.1%). The lower number of patients with later stage were collected. It may lead the unbalanced disease background between discovery and validation cohort. Due to the low incidence of *MET*ex14, it was timely unavailable to recruit more patients with *MET*ex14 advanced staged NSCLC in our center or in a multi-center approach. However, very little difference was observed in gene expression, tumor microenvironment, and signaling pathways between discovery and validation cohort. Meanwhile, constant subtype-specific biological features were observed between discovery and validation cohorts. This finding again strongly suggested the molecular subtypes were highly conservative across different stages of *MET*ex14 NSCLC. Additionally, although early-stage patients predominated in the discovery cohort, MET-Driven patients displayed advanced disease stages, aggressive clinicopathologic phenotypes and poor prognosis. Importantly, in the validation cohort, MET-driven subtype displayed a better response to MET inhibitors. Thus, transcriptomic signatures from early lung cancer patients illustrated the potential to predict outcomes of stage IV lung cancers. Secondly, only a limited number of patients received treatment of MET targeting TKIs in validation cohort. Further, frontline treatments and type of MET-TKIs used were also heterogenous. While mPFS (7.5 months) of these all 41 patients was highly comparable to published mPFS (6.9 month) of MET-TKI in Chinese NSCLC patients^50^. Highly comparable efficacy data suggested the potential bias would not largely influence the confidence of the result that MET-Driven subtype was independently associated with the clinical benefit of MET-TKIs. Nevertheless, a multi-center, prospective study is warranted to validate these preliminary results from this study. Especially, it would be more interesting to prospectively verify the potential correlation of molecular subtypes to the therapeutic efficacy of MET-TKI(s) in treatment-naïve advanced/metastatic *MET*ex14 NSCLC.

In summary, this study provided novel insight into inter-tumoral molecular heterogeneity of *MET*ex14 NSCLC. Four distinct transcriptional molecular subtypes were discovered of the first time with unique biological and clinical significance. A classifier with sufficient robustness was translated and validated in relapsed/metastatic patients for putative clinical practice. These findings together offer a novel prism for more precise therapeutic strategy for the patients with *MET*ex14 advanced/metastatic NSCLC.

## Methods

### Clinical samples

10,525 stage I-III and 3,528 stage IIIB-IV NSCLC patients receiving routine molecular diagnostics in the Department of Pathology of Shanghai Chest Hospital from 2018 to 2022 were screened. 132 *MET*ex14 skipping NSCLC surgical specimens at stage I-III (the discovery cohort) and 65 relapsed/metastatic *MET*ex14 NSCLC specimens (the independent validation cohort) were retrospectively collected. Genetic test results of *MET*ex14 were reviewed by RNA-based RT-PCR, in stage I-III patients, 3 cases did not meet the quality control requirements, 3 cases were negative, and the remaining 126 cases were positive. In relapsed/metastatic patients, 1 case did not meet the quality control requirements, and the remaining 64 cases were positive (Fig. 1, right). All tissue samples were collected in accordance with the informed consent policy. The research protocol was approved by the Ethics Committee of Shanghai Chest Hospital (No. IS2183), in accordance with the Declaration of Helsinki. All participants gave informed consent or requested waiver of informed consent prior to participation in this study. This study meets the requirements of the “Guidance of the Ministry of Science and Technology (MOST) for the Review and Approval of Human Genetic Resources”.

### DNA and RNA extraction, library construction, targeted sequencing, and data analysis

Targeted RNA sequencing covering transcripts of 2,660 onco-immunological genes and comprehensive genomic profiling in 571 cancer-related genes were performed on 126 specimens of discovery cohort (Fig. 1, left panel). Only targeted RNA sequencing was performed on 64 biopsy specimens of validation cohort (Fig. 1, right panel). Genomic DNA and RNA extraction refer to previously published literature, using AmoyDx FFPE DNA and RNA Kit (Amoy Diagnostics, Xiamen, China)^51,52^. DNA and RNA concentration was measured by Qubit (Thermo Fisher Scientific, Waltham, MA, USA). Fragment length was assessed using an Agilent 2100 Bioanalyzer and DNA HS Kit (Cat. # 5067-1504/5067-1511, Agilent). DNA was sheared into 200–250 bp fragments using Covaris LE220 (Woburn, MA, USA) and indexed NGS libraries were prepared by end repairing, A-tailing, adaptor ligation, and amplification procedures using NEBNext® Ultra™ II DNA Prep Kit (Cat. #E7645, NEB). DNA libraries were captured by AmoyDx® Master Panel, which contains 571 genes for DNA mutation (supporting single-nucleotide variation [SNV], insertion/deletion [Indel], Fusion, copy number variation, microsatellite instability and tumor mutation burden [TMB]) detection. Captured products were amplified and quantified by a Quantus fluorometer. Library size was assessed using Agilent 2100 Bioanalyzer. After pooling, libraries were then sequenced on Illumina NovaSeq 6000 instrument (Illumina) with 2 × 150 bp pair-end reads. RNA was fragmented at 95 °C for 0–15 min according to the DV200 value estimated by the Agilent 2100 Bioanalyzer System. RNA fragments then undergo reverse transcription, complementary RNA synthesis, and strand-specific library preparation using NEBNext® Ultra™ II Directional RNA Library Prep Kit for Illumina® (Cat. #E7760L, NEB). For RNA-seq, cDNA libraries were generated using a TruSeq RNA Sample Preparation kit (Illumina) according to the manufacturer’s protocol, and sequenced on Illumina NovaSeq 6000 (San Diego, CA, USA) using a panel consisting of 2660 onco-immunology genes (AmoyDx, Xiamen, China). Sequencing data were analyzed and annotated with an in-house developed pipeline. A set of experimental and data quality control parameters were set up.

### Variants calling, annotation and filtration

Single nucleotide variants (SNVs) were identified and recorded by SSBC-VarScan (version 1.3.0; AmoyDx, Xiamen, China, inhouse). Insertions/deletions (Indels) were called by Indel Caller (version 0.2.1; AmoyDx, Xiamen, China, inhouse). All variants identified were annotated with Annotator (version 0.3.6; AmoyDx, Xiamen, China, inhouse). Variants classification and criteria for filtering SNVs, Indels and fusion were provided in reference^53^.

The average depth of sequencing in the coverage area of tumor hotspots and non-hotspots was 1957.4 × and 830.5 ×, respectively. Sequencing data were first cleaned to remove sequencing adaptors and low-quality reads (quality < 15) or poly-N with Trimmomatic (Trimmomatic, RRID:SCR_011848) and mapped to the human reference genome, version 19 (hg19) using the Burrows-Wheeler Aligner. PCR duplicates were marked and removed using Mark Duplicates from the Genome Analysis Toolkit (GATK, RRID:SCR_001876). Base Quality Score Recalibration was performed using GATK’s BaseRecalibrator and ApplyBQSR. After correction, a bam file was written. The Indels and single-nucleotide polymorphisms were compared by Mutect2 and FilterMutectCalls of GATK to obtain the final vcf file. Analysis of variance was used to annotate the vcf files. SNVs and Indels called were further filtered using the following criteria^53^: (i) minimum ≥ 5 variant supporting reads and ≥ 5% variant allele frequency supporting the variant, (ii) filtered if present in > 2% population frequency in the 1000 g or ExAC or GnomAD (GnomAD, RRID:SCR_014964) database, (iii) filtered if variants not located in CDS region, (iv) filtered if variants were not annotated as (likely/predicted) oncogenic in the OncoKB database. These filtered variants were functional and were used for subsequent data analysis.

### Transcriptome annotation and gene quantification

We mapped RNA-seq paired-end reads to the Homo sapiens genome assembly GRCh37 (hg19) using STAR32 (version 020,201) with transcriptome annotation (Genecode version 20) and gene quantification was performed using RSEM 33 (version v1.2.28). Coding region reads were converted to the form of Fragments Per Kilobase Million (FPKM) to measure expressional level of each gene.

### Gene set and pathway enrichment analysis

Gene Set Enrichment Analysis (GSEA) was performed by R package clusterProfiler (v 4.2.2), with an adjusted *P* value < 0.05 as statistically significant. Gene Set Variation Analysis (GSVA) from GSVA (v 1.42.0) R package was used to evaluate enrichment scores of functional terms in GO, KEGG, Hallmark, Reactome, and several immune related signatures in NSCLC Samples.

### Consensus clustering

Log2(FPKM + 1) data matrix was first quantile normalized, followed by GSVA enrichment of terms in GO, KEGG, Hallmark, and Reactome from MsigDB database^54,55^. Consensus clustering was then utilized to identify the different subtypes by R package ConsensusClusterPlus (v 1.58.0) based on the GSVA matrix. We start with 2 consensus clusters until 6 consensus clusters build the cumulative distribution function (CDF). The optimal number of clusters is determined by both CDF and the consensus matrix.

### Mutational signatures

Mutational signatures of gene mutation from the gene panel were calculated by SigMA (v 1.6) R package, in which signatures 1 to 30 were obtained from the COSMIC database (https://cancer.sanger.ac.uk/signatures/signatures_v2/), and signatures APOBEC and smoking were clustered based on the COSMIC signatures^56^. Somatic SNV and small deletion and insertion, were included for calculation.

### T-Distributed Stochastic Neighbor Embedding (t-SNE)

Applying the unsupervised nonlinear dimensionality reduction method t-SNE^57^, how tumors in the discovery cohort were localized to each other was investigated in the dimensionality reduction space based on CNVs that differed significantly among the four subtypes.

### Differential gene expression and pathway enrichment

Analysis of differential gene expression (DEG) in each subtype was performed using Limma (v 3.50.0) package, and different pathway enrichment score was analyzed by Wilcoxon test, with false discovery rate (FDR) < 0.05 as threshold^58^.

### In silico Tumor microenvironment characterization & Deconvolution of infiltrated immune cells

Several TME signatures, including 29 TME gene expression signatures^59^, 28 immune gene sets^60^, Danaher^61^, and AO^62^ were scored by GSVA. MCPcounter^63^ and xCell score^64^ was analyzed by R packages MCP counter (v 1.2.0) and xCell (v 1.1.0), respectively.

### Classifier construction

Univariate logistic regression was performed for each cluster in the sample set of 126 stage I-III NSCLC patients. The top 10 genes with the highest odds ratio (OR) values and the bottom 10 genes with the lowest OR values were selected for classifier construction. 39 genes were screened out via Lasso-logistic regression and a multivariable logistic regression classifier was constructed. The classifier was further validated internally by 200 iterations of 5-fold cross-validation and leave-one-out cross-validation. The molecular subtype was predicted by maximal likelihood given by this trained classifier model in 64 relapsed/metastatic NSCLC samples.

### RT-PCR analysis for *MET*ex14 transcripts

RNA was converted to cDNA using M-MLV retro-transcriptase (Thermo Fisher Scientific) and oligo-dT primers, and *MET*ex14 was amplified using Hot-Start Taq polymerase (Qiagen) in a 20 μL reaction and visualized in agarose gels. Primers sequences were as follows: forward (exon 13) 5’-TTTTCCTGTGGCTGAAAAAGA-3’ and reverse (exon 15) 5’-GGGGACATGTCTGTCAGAGG-3’. Amplification generated a 246-bp band for wild-type (WT) *MET* RNA and a 106-bp band for *MET*ex14. Positivity for *MET*ex14 was determined using a real-time reverse transcription-polymerase chain reaction (RT-PCR) assay (AmoyDx, Xiamen, China) performed at laboratories.

### Multiplex immunofluorescence

Multiplex immunofluorescence (mIF) staining was performed to visualize the expression of CD8, GZMK, PD-L1, CD4, CK, CD20 and FOXP3 in tumor tissues. Three consecutive sections (3 μm-thick) were obtained from paraffin blocks. One section was used for H&E staining and the other two sections were used for mIF staining^65^. The primary antibodies used for each staining were Rabbit monoclonal CD4 (Abcam Cat# ab133616, RRID: AB_2750883), Rabbit monoclonal CD8 alpha (Abcam Cat# ab245118, RRID: AB_3068617), Rabbit monoclonal Granzyme K (Abcam, ab282703), Rabbit monoclonal PD-L1 (Abcam Cat# ab213524, RRID: AB_2857903), Mouse monoclonal Cytokeratin (Long Island Antibody, #349), Rabbit monoclonal CD20 (Abcam Cat# ab78237, RRID: AB_1640323), Mouse monoclonal FOXP3 (Abcam Cat# ab20034, RRID: AB_445284). The concentration and staining order of the antibodies used in this study were optimized beforehand. After all sequential staining steps, nuclei acids were stained with DAPI.

### Statistical analysis

Demographic characteristics and mutational landscape of patients were analyzed using descriptive statistics. Chi-square or Fisher’s exact probability tests were performed when the rate or percentage was compared for significance. Nonparametric Wilcoxon rank sum tests were used for the comparison of medians between the two datasets. ANOVA tests were utilized for the comparison among three or more groups. Kaplan–Meier survival analysis was used to evaluate the association between subtypes and progression-free survival. Survival curves were compared by using the log-rank test. A multivariable logistic regression model based on differentiated expressed genes in each subtype was used to build a subtype classifier. k-fold cross-validation and leave-one-out cross-validation were used to determine subtype classifier performance. Univariate and multivariate Cox regression analysis was performed to figure out the potential risk factors. All statistical analyses were performed with the R software (R Project for Statistical Computing, RRID:SCR_001905). *P* value < 0.05 was considered statistically significant.

### Supplementary information

Supplementary information

## Data Availability

The raw sequencing data generated in this study have been deposited in the GSA for Human (Genome Sequence Archive for Human in BIG Data Center, Beijing Institute of Genomics, Chinese Academy of Sciences, https://ngdc.cncb.ac.cn/gsa-human/) under the accession code HRA007761. Any additional information required to reanalyze the data reported in this paper is available from the corresponding authors upon request.

## Code availability

This paper does not report the original code.
