# Supplementary material for: Novel molecular subtypes of METex14 non-small cell lung cancer with distinct biological and clinical significance
Source: NPJ Precis Oncol. 2024 Jul 26;8:159. doi: 10.1038/s41698-024-00642-6 (PMC11282101; doi:10.1038/s41698-024-00642-6)
Supplement: Supplementary file 1 — Supplementary information [file 41698_2024_642_MOESM1_ESM.pdf]

Novel molecular subtypes of METex14 non-small cell lung cancer with distinct biological and clinical significance

Supplementary Materials

Supplementary Tables

Supplementary Table 1. Baseline characteristics of relapsed/metastatic NSCLC patients with METex14 in the independent validation cohort

Characteristic	Overall (n = 64)	MET-Driven (Subtype A) (n = 44)	FGFR-Activated (Subtype B) (n = 2)	Immune-Activated (Subtype C) (n = 14)	Bypass-Activated (Subtype D) (n = 4)	P-value
**Gender**						
Female	20 (31.3%)	14 (31.8%)	0 (0%)	4 (28.6%)	2 (50.0%)	0.817
Male	44 (68.8%)	30 (68.2%)	2 (100%)	10 (71.4%)	2 (50.0%)	
**Age**						
Mean (SD)	72.3 (7.66)	72.8 (7.79)	74.5 (9.19)	70.2 (6.31)	72.0 (11.5)	0.675
Median [Min, Max]	73.0 [56.0, 90.0]	73.5 [56.0, 90.0]	74.5 [68.0, 81.0]	71.0 [59.0, 82.0]	74.5 [57.0, 82.0]	
**Pathological Type**						
Adeno	36 (56.3%)	23 (52.3%)	1 (50.0%)	10 (71.4%)	2 (50.0%)	0.695
NSCLC	16 (25.0%)	12 (27.3%)	0 (0%)	3 (21.4%)	1 (25.0%)	
Squamous	12 (18.8%)	9 (20.5%)	1 (50.0%)	1 (7.1%)	1 (25.0%)	

Supplementary Table 1. Baseline characteristics of relapsed/metastatic NSCLC patients with METex14 in the independent validation cohort

Characteristic	Overall (n = 64)	MET-Driven (Subtype A) (n = 44)	FGFR-Activated (Subtype B) (n = 2)	Immune-Activated (Subtype C) (n = 14)	Bypass-Activated (Subtype D) (n = 4)	P-value
**Lymph Node Metastasis**						
No	59 (92.2%)	41 (93.2%)	1 (50.0%)	13 (92.9%)	4 (100%)	0.282
Yes	5 (7.8%)	3 (6.8%)	1 (50.0%)	1 (7.1%)	0 (0%)	
**Malignant Pleural Effusion**						
No	60 (93.8%)	42 (95.5%)	1 (50.0%)	14 (100%)	3 (75.0%)	0.0459
Yes	4 (6.3%)	2 (4.5%)	1 (50.0%)	0 (0%)	1 (25.0%)	
**Distal Metastasis Lung**						
No	59 (92.2%)	40 (90.9%)	2 (100%)	14 (100%)	3 (75.0%)	0.353
Yes	5 (7.8%)	4 (9.1%)	0 (0%)	0 (0%)	1 (25.0%)	
**Brain**						
No	56 (87.5%)	39 (88.6%)	2 (100%)	12 (85.7%)	3 (75.0%)	0.734
Yes	8 (12.5%)	5 (11.4%)	0 (0%)	2 (14.3%)	1 (25.0%)	
**Bone**						
No	42 (65.6%)	27 (61.4%)	2 (100%)	10 (71.4%)	3 (75.0%)	0.699
Yes	22 (34.4%)	17 (38.6%)	0 (0%)	4 (28.6%)	1 (25.0%)	

Supplementary Table 1. Baseline characteristics of relapsed/metastatic NSCLC patients with METex14 in the independent validation cohort

Characteristic	Overall (n = 64)	MET-Driven (Subtype A) (n = 44)	FGFR-Activated (Subtype B) (n = 2)	Immune-Activated (Subtype C) (n = 14)	Bypass-Activated (Subtype D) (n = 4)	P-value
**Pleura**						
No	57 (89.1%)	39 (88.6%)	2 (100%)	12 (85.7%)	4 (100%)	1
Yes	7 (10.9%)	5 (11.4%)	0 (0%)	2 (14.3%)	0 (0%)	
**Vertebral**						
No	61 (95.3%)	41 (93.2%)	2 (100%)	14 (100%)	4 (100%)	1
Yes	3 (4.7%)	3 (6.8%)	0 (0%)	0 (0%)	0 (0%)	
**Adrenal gland**						
No	60 (93.8%)	41 (93.2%)	2 (100%)	13 (92.9%)	4 (100%)	1
Yes	4 (6.3%)	3 (6.8%)	0 (0%)	1 (7.1%)	0 (0%)	
**Multiple site**						
No	49 (76.6%)	33 (75.0%)	1 (50.0%)	12 (85.7%)	3 (75.0%)	0.607
Yes	15 (23.4%)	11 (25.0%)	1 (50.0%)	2 (14.3%)	1 (25.0%)	

Supplementary Table 2. Baseline characteristics of patients receiving MET targeted therapy in the independent validation cohort

Characteristic	Overall (n = 41)	MET-Driven (n = 26)	Others (n = 15)	P-value
**Age**				
Mean (SD)	70.3 (8.05)	71.2 (8.62)	68.8 (6.99)	0.448
Median [Min, Max]	69.0 [59.0, 90.0]	70.0 [60.0, 90.0]	68.0 [59.0, 82.0]	
**Gender**				
Female	14 (34.1%)	8 (30.8%)	6 (40.0%)	0.734
Male	27 (65.9%)	18 (69.2%)	9 (60.0%)	
**Smoking**				
Never	6 (14.6%)	4 (15.4%)	2 (13.3%)	1
Ever/Current	16 (39.0%)	9 (34.6%)	7 (46.7%)	
NA	19 (46.3%)	13 (50.0%)	6 (40.0%)	
**Pathological Stage***				
III	9 (22.0%)	5 (19.2%)	4 (26.7%)	0.701
IV	32 (78.0%)	21 (80.8%)	11 (73.3%)	
**Treatment**				
Capmatinib	3 (7.3%)	2 (7.7%)	1 (6.7%)	0.642
Crizotinib	16 (39.0%)	9 (34.6%)	7 (46.7%)	
Glumetinib+Osimertinib	1 (2.4%)	1 (3.8%)	0 (0%)	
Savolitinib	20 (48.8%)	14 (53.8%)	6 (40.0%)	

Supplementary Table 2. Baseline characteristics of patients receiving MET targeted therapy in the independent validation cohort

Characteristic	Overall (n = 41)	MET-Driven (n = 26)	Others (n = 15)	P-value
Salvolitinib + Crizotinib	1 (2.4%)	0 (0%)	1 (6.7%)	
**Treatment lines**				
First line	23 (56.1%)	11 (42.3%)	12 (80.0%)	0.0631
Second line	17 (41.5%)	14 (53.8%)	3 (20.0%)	
Third line	1 (2.4%)	1 (3.8%)	0 (0%)	

Abbreviations: NA, not applicable; NSCLC: Non-Small Cell Lung Cancer;

*: American Joint Committee on Cancer (AJCC) 8th

Supplementary Figures

Supplementary Figure 1

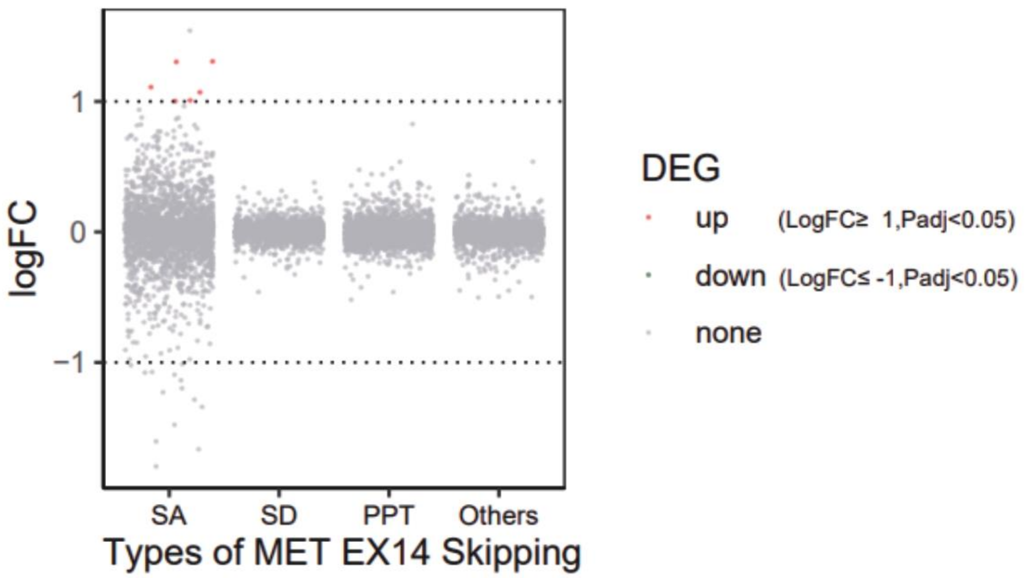

Supplementary Figure 1. Differentiated Expressed genes across various genomic locations of MET exon 14 skipping.

Supplementary Figure 2

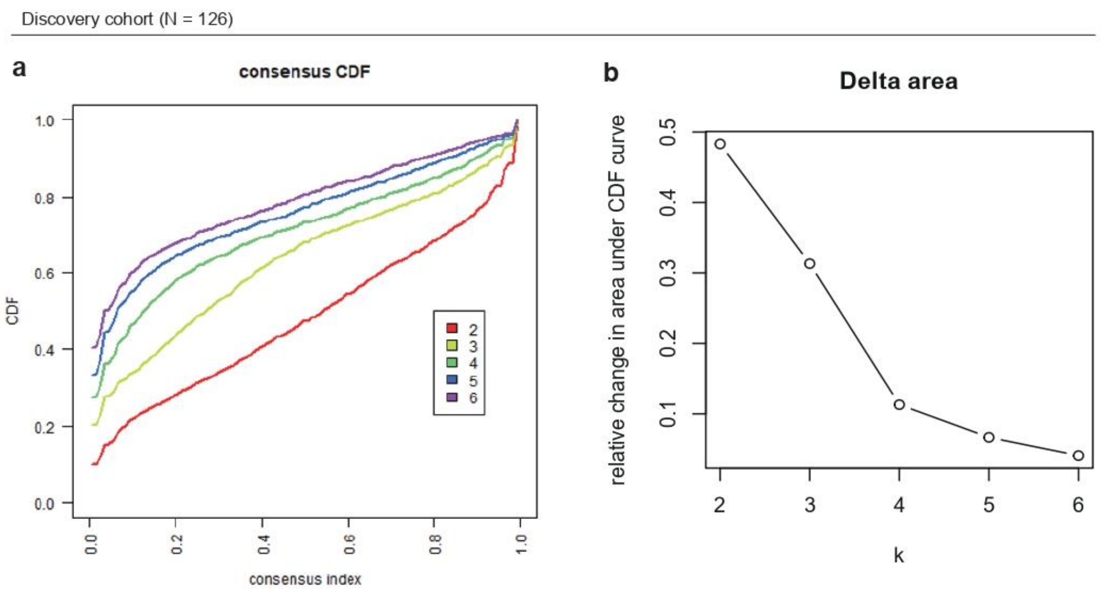

Supplementary Figure 2. Determining Optimal cut-off for k-means clustering. (a) The best cluster counts (k = 4) estimation for clustering METex14 NSCLC from the discovery cohort based on RNA-seq data. (b) Relative change in area under CDF curve for k = 4.

Supplementary Figure 3

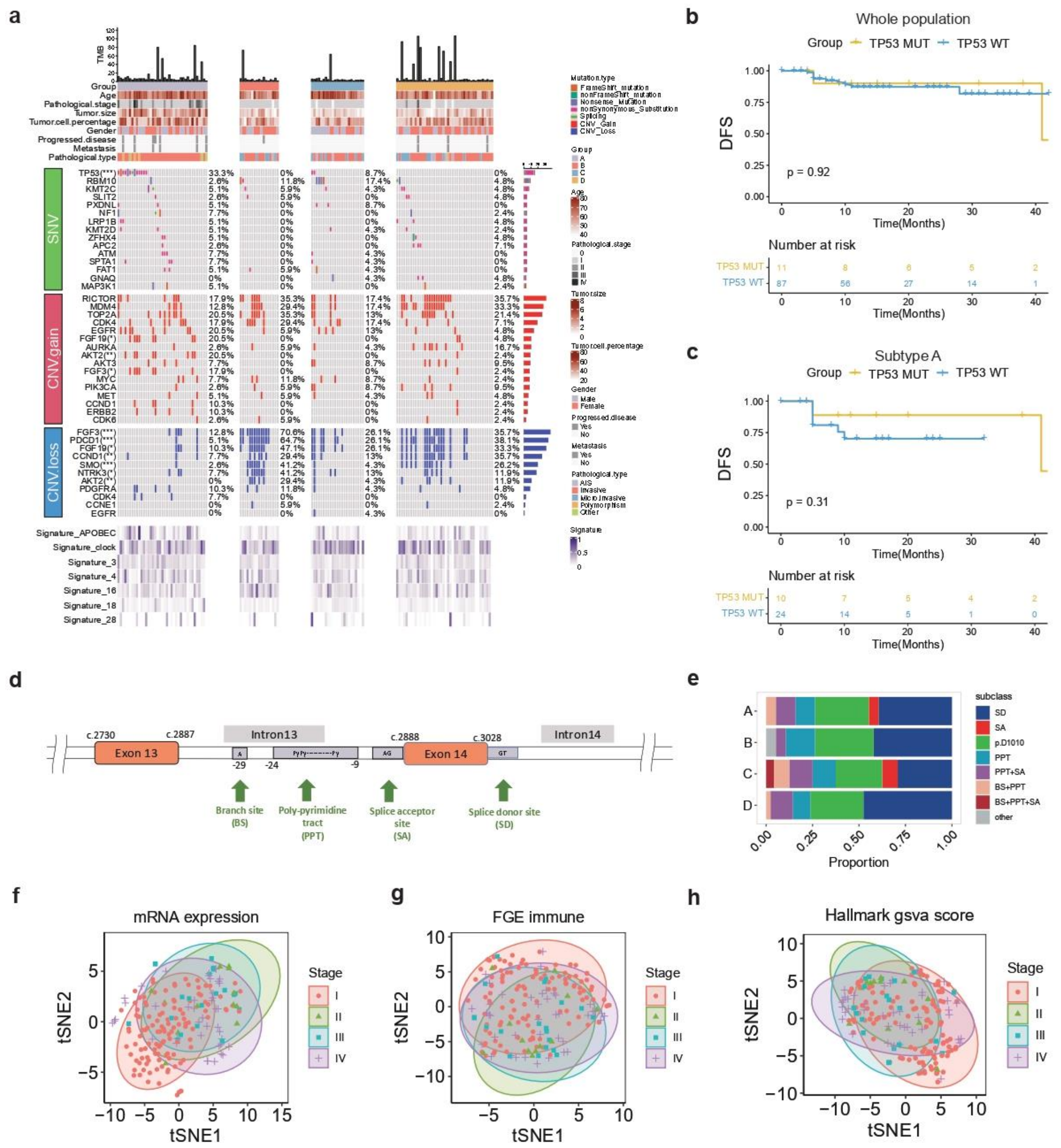

Supplementary Figure 3. Mutational landscape of the four subtypes from the discovery cohort. (a) Gene mutation status, Genes with somatic mutations were listed on the y-axis, and samples were shown on the x-axis, mutation frequencies of each gene were shown on the right, the corresponding clinical features of individual patients are presented at the top of (a), the enrichment score of each COSMIC signature profile are presented at the bottom of (a). DFS of patients with *TP53* mutations and *TP53* WT patient in the whole discovery cohort (b) and the subtype A (c). (d) Schematic

illustration of the spectrum of MET exon 14 skipping mutations. (e) Distribution of Genomic locations of MET exon 14 skipping mutations across four molecular subtypes. The difference of the mRNA expression (f), tumor microenvironment (g), and the signaling pathways enrichment (h) among stage I to IV patients in the discovery cohort.

Supplementary Figure 4

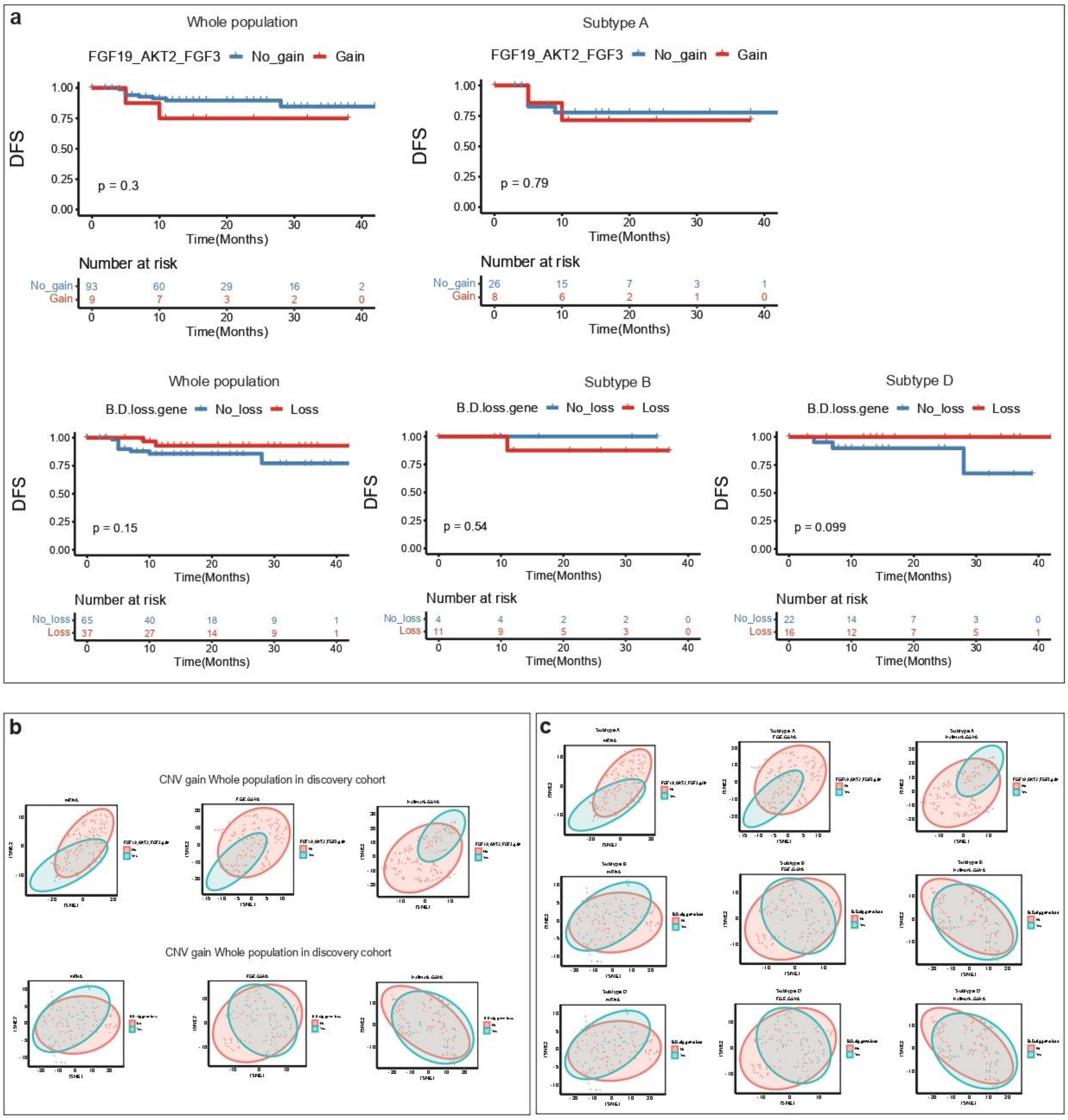

Supplementary Figure 4. (a) DFS of patients with CNV gain of *FGF19*, *FGF3*, or *AKT2*, and CNV loss of *FGF19*, *FGF3*, *AKT2*, *NTRK3*, *PDCD1*, *CCND1*, *or SMO* in the whole discovery cohort and subtype A, subtype B, and subtype D. (b) The difference of the mRNA expression, tumor microenvironment, and the signaling pathways enrichment between with CNV gain and with CNV loss patients in the whole discovery cohort. (c) The difference of the mRNA expression, tumor microenvironment, and the

signaling pathways enrichment between with CNV gain and with CNV loss patients among subtype A, subtype B, and subtype D patients in the discovery cohort.

Supplementary Figure 5

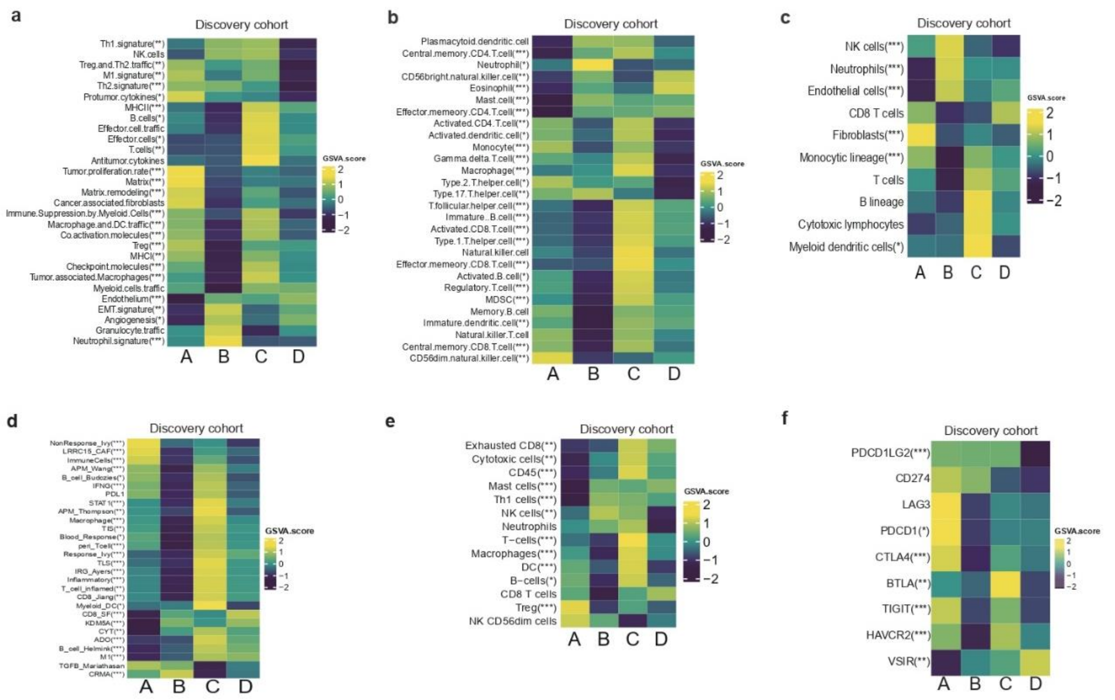

Supplementary Figure 5. Detailed GSVA analysis results of 29immune (a), 28immune (b), MCPCounter (c), AO (d), and Danaher (e) signature. (f) GSVA analysis results of detailed gene expression of “checkpoint molecules” in 29immune signature. Data from the discovery cohort.

Supplementary Figure 6

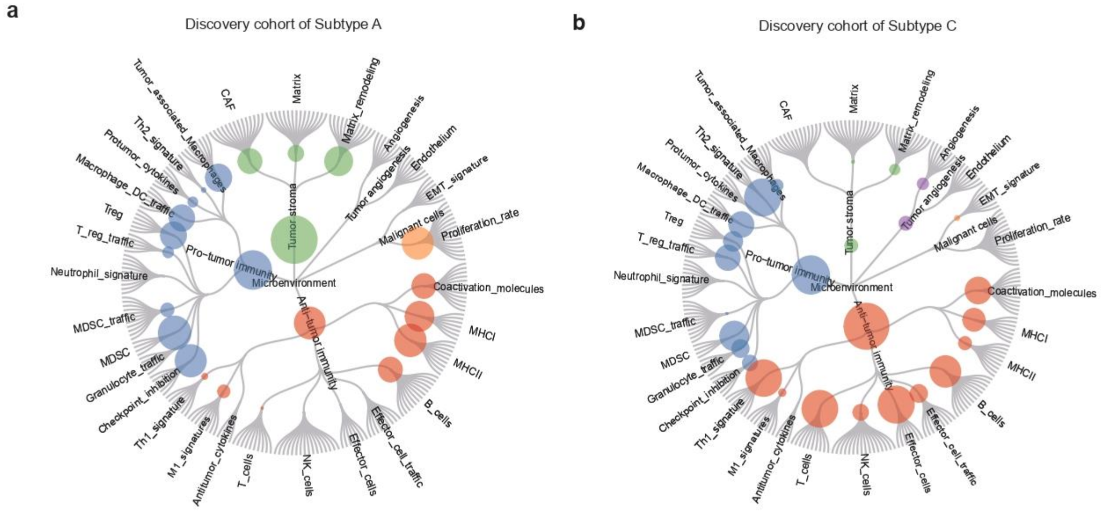

Supplementary Figure 6. (a) Molecular Functional Portrait (potential targetable genes, signaling pathways, and cellular processes related to each of 29 TME gene expression signatures created by Bagaev et al.) of subtype A. (b) Molecular Functional Portrait of subtype C.

Supplementary Figure 7

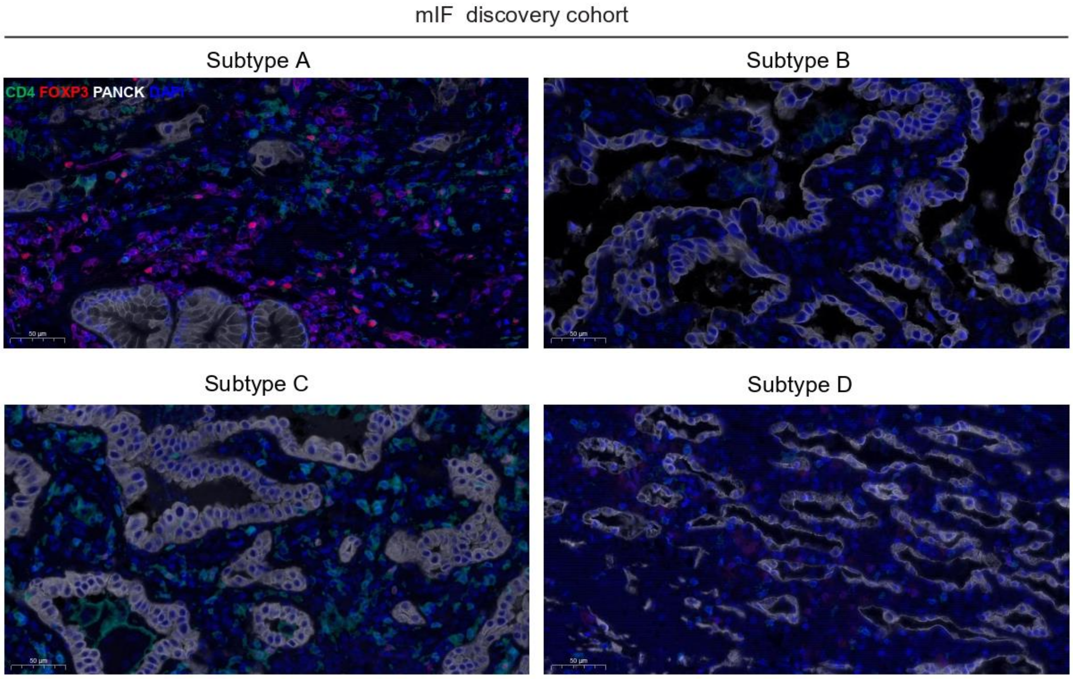

Supplementary Figure 7. Representative examples of mIF of Treg cells for each of the 4 subtypes in the discovery cohort.

Supplementary Figure 8

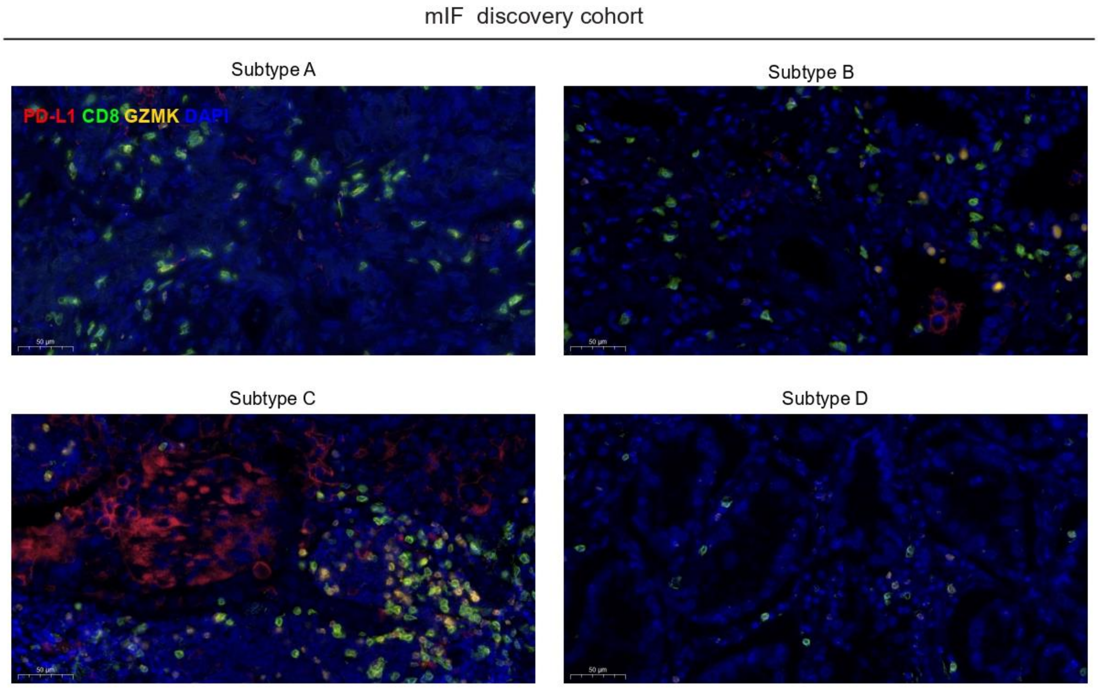

Supplementary Figure 8. Representative examples of mIF of effector CD8+ T cells for each of the 4 subtypes in the discovery cohort.

Supplementary Figure 9

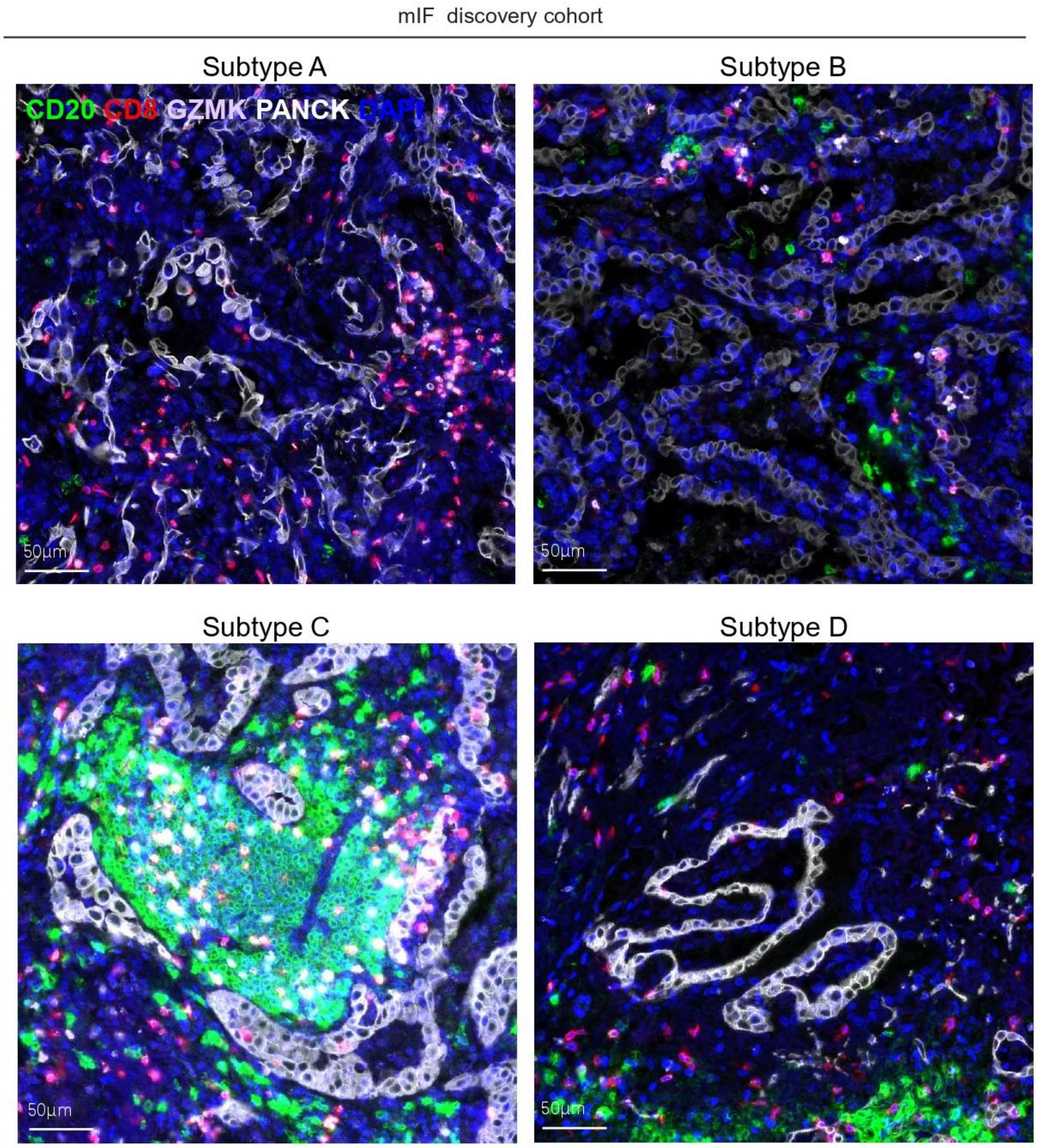

Supplementary Figure 9. Representative examples of mIF of CD20+ B cells for each of the 4 subtypes in the discovery cohort.

Supplementary Figure 10

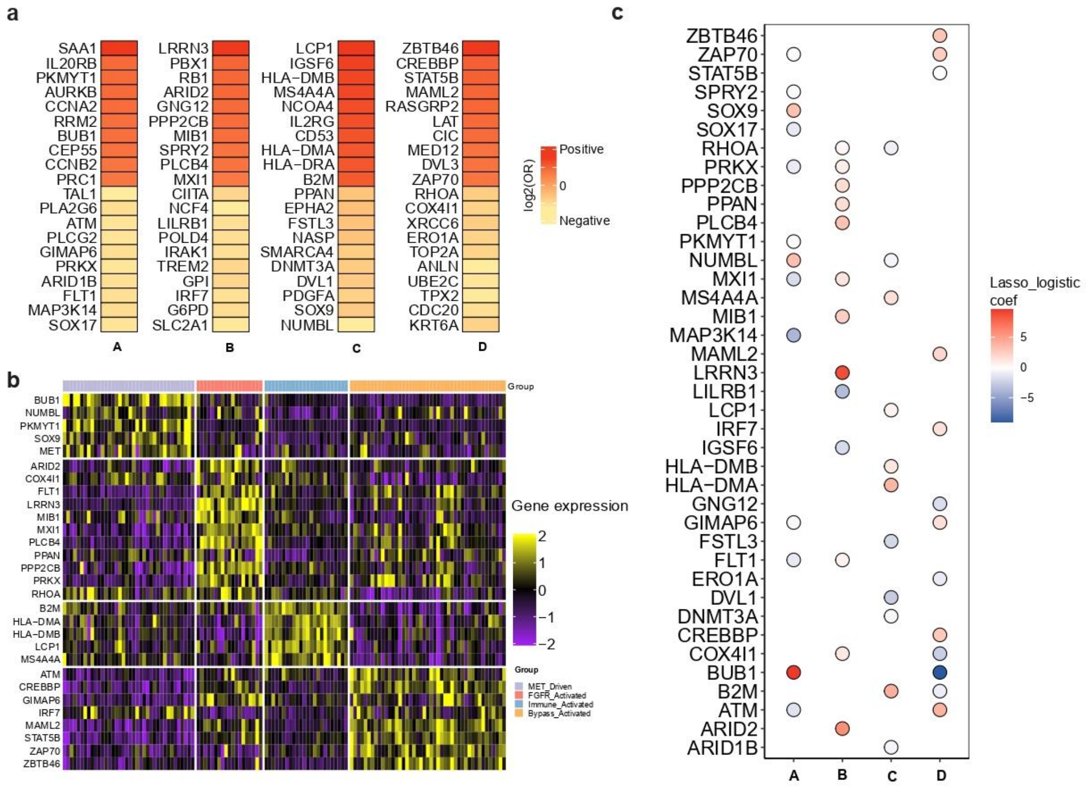

Supplementary Figure 10. (a) 20 genes were screened for each subtype using univariant logistic regression. (b) 39 genes were screened by Lasso-logistic for model construction. (c) Heatmap of the expression of the 39 genes and *MET* across the four subtypes.

Supplementary Figure 11

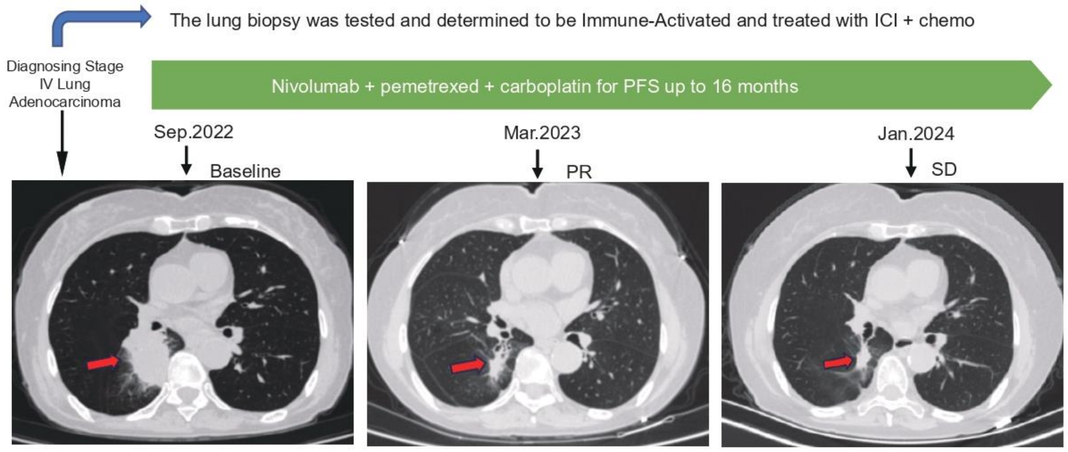

Supplementary Figure 11. A 68-year-old female, non-smoker was diagnosed as stage IV lung adenocarcinoma with MET Exon 14 skipping. This patient was classified as Immune Active subtype. The patient was then treated with pemetrexed plus carboplatin (AC) combined with nivolumab and achieved partial response (PR) with a progression-free survival (PFS) of 16 months.
